# Evaluation of nurses’ perception of monkeypox in terms of epidemic anxiety, stress levels and compliance with isolation measures

**DOI:** 10.1186/s12912-025-03530-x

**Published:** 2025-07-08

**Authors:** Vildan Kocatepe, Dilek Yildirim, Ayşegül Türkmenoğlu

**Affiliations:** 1 Department of Nursing, Faculty of Health Sciences, Izmir Demokrasi University, Izmir, Turkey; 2 Department of Nursing, Faculty of Health Sciences, Istanbul Aydin University, Istanbul, Turkey; 3 Izmir City Hospital, Izmir, Turkey

**Keywords:** Anxiety, Compliance, Monkeypox, Nurses, Stress

## Abstract

**Backgrounds:**

Understanding how nurses perceive anxiety and stress during an epidemic is essential for curbing the spread of disease and implementing effective public health strategies. This study aims to evaluate the levels of epidemic anxiety, perceived stress, and compliance with isolation measures among nurses working in Turkey in relation to monkeypox.

**Methods:**

This descriptive, cross-sectional study was conducted with a sample of 335 nurses employed at a hospital in Izmir, Turkey. Data were collected through face-to-face interviews between September 15 and November 30, 2024, using a structured questionnaire. The data collection instruments included a Demographic Information Form, the Epidemic Anxiety Scale, the Perceived Stress Scale, and the Compliance with Isolation Precautions Scale.

**Results:**

The mean age of the nurses was 28.41 ± 6.26 years. The average total score was 79.37 ± 10.01 on the Compliance with Isolation Precautions Scale, 49.16 ± 15.44 on the Epidemic Anxiety Scale, and 26.39 ± 7.76 on the Perceived Stress Scale. Statistically significant differences in the total scores of the Compliance with Isolation Precautions Scale were observed based on gender, prior experience working in a pandemic-designated hospital, and current department of employment (*p* <.005). A statistically significant but weak negative correlation was found between the Compliance with Isolation Precautions Scale and both the Epidemic Subscale (*r* = −.124; *p* =.031) and the Economic Subscale (*r* = −.129; *p* =.023). Additionally, a statistically significant moderate positive correlation was identified between the Perceived Stress Scale and the Epidemic Anxiety Scale (*r* =.399; *p* <.001).

**Conclusion:**

The study revealed that nurses experienced a moderate level of epidemic-related anxiety. Moreover, it was found that a substantial majority of nurses (95.8%) had not received any formal training on monkeypox. The findings also indicated that nurses’ perceived stress levels increased in parallel with their levels of epidemic anxiety.

## Background

Human monkeypox (Mpox) is a contagious disease caused by the monkeypox virus, which is rarely fatal. Although the virus was first identified in monkeys in 1958, the initial human cases were reported in the 1970s. Mpox and smallpox viruses both belong to the Poxviridae family. These viruses typically cause infections through zoonotic transmission, i.e., from animals to humans. On August 14, 2024, the World Health Organization (WHO) declared the Mpox outbreak a Public Health Emergency of International Concern (PHEIC), citing its rapid global spread and potential public health threat [1].

According to a statement released by the World Health Organization (WHO), a total of 15,600 Mpox cases and 537 associated deaths were reported in 2024 alone. One day prior to this announcement, the existing public health emergency—previously designated at the continental level—was elevated to a global status due to the increasing international spread and severity of the outbreak. The Mpox virus has since been identified in at least 13 African countries, including Burundi, Kenya, Rwanda, and Uganda, which had not reported cases in previous years. The Democratic Republic of the Congo (DRC) remains the most heavily affected country. Across the African continent, the number of suspected cases has surged, with 7,146 cases reported in 2022 and 14,957 in 2023. In its situation report dated August 12, 2024, WHO documented a total of 99,176 confirmed cases and 208 deaths across 116 countries between January 1, 2022, and June 30, 2024. Notably, 96% of these cases were reported from the DRC. As of 2024, Mpox has emerged as a growing global health threat, with increasing rates of cross-border transmission [1–4]. Mpox cases drew significant global attention in 2022 and 2023, particularly following the World Health Organization’s declaration of a global health emergency. In response, the Turkish Ministry of Health issued comprehensive guidelines for the diagnosis and management of the disease. An official statement released on August 15, 2024, confirmed that no Mpox cases were reported in Turkey during that year and emphasized that all necessary preventive measures had been implemented. According to data from the U.S. Centers for Disease Control and Prevention (CDC), a total of 12 Mpox cases have been reported in Turkey since 2022 [5]. However, recent surveillance data indicate that no new cases have been reported in the past four ISO weeks or the last three months [6].

The Mpox virus can be transmitted from infected animals to humans, as well as through human-to-human transmission via multiple routes. The primary mode of transmission is direct contact with the bodily fluids, skin lesions, or scabs of infected individuals. Additionally, the virus can be transmitted through skin-to-skin contact, making such contact one of the most significant risk factors for infection [5, 7, 8]. The virus may also spread through respiratory droplets, particularly in cases of prolonged and close contact. Furthermore, transmission can occur through activities such as kissing, hugging, and contact with contaminated clothing or bedding. Vertical transmission from a pregnant woman to her fetus via the placenta is also possible. Consequently, individuals in indoor or crowded settings, or those in close contact with infected persons, are considered to be at elevated risk [5, 9, 10].

Nurses, as a vital component of the healthcare workforce and one of its largest groups, play a key role in counseling patients and their caregivers to prevent the transmission of infectious diseases. This counseling extends beyond the provision of medical care; nurses have a crucial responsibility in safeguarding public health and curbing the spread of infections. Their knowledge, attitudes, and practices significantly impact not only patient outcomes but also the effective control of infectious disease outbreaks at large [11–13]. Nurses, who are at the forefront of direct patient care, maintain continuous interaction with patients and other healthcare professionals to prevent the spread of infections. Therefore, for infection control strategies to be effective, it is essential that nurses possess accurate and up-to-date knowledge about the nature of infections and their transmission routes. Beyond basic treatment, nurses enhance the effectiveness of healthcare systems by taking on an educational role, promoting proper hygiene and preventive measures. Moreover, their careful attention before, during, and after infection can have a profound impact on both individual patient outcomes and public health as a whole [10, 12, 13].

While many factors influence individuals’ stress and anxiety levels, health-related issues tend to have the most significant impact. Life-threatening health conditions can profoundly alter a person’s psychological state. Understanding how a health problem—especially one associated with mortality risk—affects stress and anxiety levels is essential for implementing appropriate preventive measures. Moreover, the mere possibility of a rapidly spreading epidemic can disrupt societal order and profoundly affect individuals’ daily lives [13, 14]. The spread of fear and panic within the community can be a major barrier to controlling an epidemic. Therefore, gaining an accurate understanding of anxiety and stress experienced during an outbreak is crucial both for disease containment and the effective implementation of public health measures. Since nurses and healthcare workers maintain continuous contact with infected patients during pandemics, they must manage not only the risk of transmission but also the professional stress associated with their roles [15]. Especially in the aftermath of a pandemic like COVID-19, it remains uncertain whether nurses are adequately prepared to face diseases such as Mpox—where diagnosis criteria are still unclear and information about risk factors and transmission routes is limited—as well as how they cope with the associated levels of anxiety and stress [13]. Although there are currently no confirmed Mpox cases in Turkey, the increasing frequency of emerging infectious diseases underscores the importance of proactive preparedness measures. The global transmission potential of Mpox, coupled with the psychological vulnerability observed among healthcare workers following the COVID-19 pandemic, warrants the examination of anxiety, stress, and preparedness-even in countries without active cases. Understanding nurses’ psychological and behavioral responses prior to an outbreak can provide valuable insights for early public health planning, targeted preparedness training, and the reinforcement of healthcare system resilience. This study aims to evaluate the levels of epidemic anxiety, perceived stress, and compliance with isolation measures among nurses working in Turkey in relation to Mpox.

## Methods

### Research design

This is a descriptive, cross-sectional study. The study was conducted with 335 nurses working in a city hospital in Izmir, Turkey between September and November 2024.

### Population and sample

The population of the study consisted of all nurses (*N* = 2200) working in a hospital in Izmir. The sample size of the study was calculated according to the known sample calculation [N = Nt^2^ pq /d^2^ (N-1) + t^2^ pq]. The reliability level was taken as 90%, standard deviation as 0.05, and margin of error as 5%. The value of *p* was assumed to be 0.5. The minimum sample size of the study was determined as 241 nurses. The study was completed with 335 nurses who agreed to participate in the study, had been working in the hospital for at least one month, and responded to all questions. Nurses with communication problems, psychiatric health problems, and nurses who were on leave on the dates of data collection were excluded from the study.

### Data collection

Data were collected through face-to-face interviews using a questionnaire between September 15 and November 30, 2024. Completing the questionnaire took approximately 20 min. The instruments used included a Participant Information Form, the Epidemic Anxiety Scale, the Perceived Stress Scale, and the Compliance with Isolation Precautions Scale. To minimize potential measurement bias, all data collection was conducted directly by researchers who had undergone comprehensive training on standardized data collection procedures. During this training, researchers were instructed to emphasize confidentiality and anonymity to participants, assuring them that their responses would remain strictly anonymous and would not impact their professional standing. Additionally, researchers were trained to maintain a neutral attitude and avoid any verbal or non-verbal cues that might influence participants’ responses. These measures were implemented to reduce social desirability bias and enhance the reliability of the self-reported data. The primary dependent variable was the Epidemic Anxiety Scale score, while the main outcome variable was the Compliance with Isolation Precautions Scale score. Independent variables included socio-demographic and professional characteristics such as age, gender, educational level, department of employment, prior experience working in a pandemic hospital, chronic disease diagnosis, training on Mpox, and Perceived Stress Scale scores.

#### Participant information form

This form was created by the researchers in line with the literature. It is a form consisting of 10 questions including sociodemographic data such as age, gender, educational status, etc., working status and characteristics of nurses related to infectious diseases [10].

#### Epidemic Anxiety Scale

The Epidemic Anxiety Scale developed by Sayar et al. in 2020. The scale measures the anxiety levels of individuals regarding epidemic, economic, quarantine and social life dimensions [16]. This five-point Likert-type scale consists of 18 items divided into four subscales: ‘Epidemic,’ ‘Economic,’ ‘Quarantine,’ and ‘Social Life.’ The total score ranges from 18 to 90, with higher scores indicating greater levels of epidemic-related anxiety. Cronbach’s alpha reliability for the entire scale is reported as 0.90, while subscale values range between 0.78 and 0.89 [16]. In the present study, the scale demonstrated excellent internal consistency, with a Cronbach’s alpha of 0.937. Total scores are interpreted as follows: 18–32 indicates “no anxiety,” 33–46 “low anxiety,” 47–61 “moderate anxiety,” 62–75 “high anxiety,” and 76–90 “very high anxiety.”

#### Perceived stress scale

This scale was developed by Cohen, Kamarck, Mermelstein [17] and adapted into Turkish by Eskin et al. [18]. The scale is designed to measure the degree of stress perceived by the individual in various situations in his/her life. Possible scores range from 0 to 56. Higher scores indicate higher stress perception. The internal consistency coefficient values for the whole scale and the self-efficacy and stress perception sub-dimensions are 0.84, 0.82 and 0.66, and the test-retest reliability coefficients are 0.87, 0.88 and 0.72, respectively [18]. The Cronbach’s alpha internal consistency coefficient of the scale was 0.875 in this study.

#### Compliance with Isolation Precautions Scale

The scale was developed by Tayran and Ulupınar in Turkish to evaluate compliance with isolation measures [19]. The scale consists of 18 items, and exploratory factor analysis identified four subdimensions: ‘Route of Infection,’ ‘Practitioner-Patient Safety,’ ‘Environmental Safety,’ and ‘Hand Hygiene/Glove Use.’ Items are rated on a 5-point Likert scale. Total scores range from 18 to 90, with higher scores indicating better adherence to isolation precautions. Regarding reliability, the Cronbach’s alpha coefficient for the entire 18-item scale was 0.85. The internal consistency coefficients for the subscales were as follows: ‘Route of Infection’ 0.80, ‘Practitioner-Patient Safety’ 0.72, ‘Environmental Safety’ 0.61, and ‘Hand Hygiene/Glove Use’ 0.52 [19]. The Cronbach’s alpha internal consistency coefficient of the scale was 0.849 in this study.

### Data analysis

Data analysis was performed using SPSS version 26.0. Socio-demographic characteristics of the participants were analyzed descriptively. To assess the normality of the data, both the Kolmogorov–Smirnov and Shapiro–Wilk tests were conducted using multiple normality methods. Descriptive statistics were presented as counts, percentages, and means. Continuous variables were summarized using means, standard deviations, and minimum–maximum values. Pearson’s correlation test was used to examine relationships between mean scores of the Epidemic Anxiety Scale, Perceived Stress Scale, and Compliance with Isolation Precautions Scale. For comparisons between two groups, Student’s t-test was applied to normally distributed quantitative variables, while the Mann–Whitney U test was used for variables not normally distributed. For comparisons among more than two groups, One-Way Analysis of Variance (ANOVA) was employed for normally distributed variables, and the Kruskal–Wallis test was used for non-normally distributed variables. A p-value of less than 0.05 was considered statistically significant.

### Ethical considerations and informed consent

#### Ethical approval

from the Non-Interventional Ethics Committee of Izmir City Hospital was obtained (Date: 11.09.2024; Ethics Approval Number:2024/136). Permission was obtained from Izmir Provincial Health Directorate. In addition, detailed information about the study was provided to the nurses who formed the sample of the study. Informed consent was obtained from all participants prior to their inclusion in the study. The nurses were also informed of their rights to refuse participation and withdraw from the study at any time.

Access to the collected data was restricted solely to the research team. To ensure data security, all electronic data were stored on a password-protected computer, while physical documents were kept in a locked cabinet. Participation consent forms and research data collection forms were stored in separate folders, making it impossible to link responses to specific individuals, thereby ensuring full anonymity. Furthermore, all data will be securely deleted and destroyed after the completion of the study in accordance with ethical guidelines. If deemed necessary, anonymized data can be shared with the journal upon request. The research was conducted in accordance with the Declaration of Helsinki.

## Results

The ages of the nurses ranged from 22 to 53, with a mean age of 28.41 ± 6.26. Most of the nurses were female (78.2%), and most had a bachelor’s degree (79.4%). 83.6 of the nurses did not have a chronic disease. The total number of years of work of the nurses ranged from 6 months to 31 years, with a mean of 4.78 ± 6.25 years. 105 (31.3%) of the nurses worked in the intensive care unit, 39 (11.6%) in the emergency department, and 154 (46.0%) in other inpatient services. 230 (68.7%) of the nurses didn’t work in a pandemic hospital before. 321 (%) of the participants didn’t receive training on Mpox before (Table 1).

**Table 1:** Socio-demographic data of the participants (*n* = 335)

Charecteristics		Mean ± SD	
Age (years)		28.41 ± 6.26	
Duration of Professional Experience (years)		4.78 ± 6.25	
**Parameters**		**n**	**%**
**Gender**	Female	262	78.2
	Male	73	21.8
**Educational Level**	Vocational High School	24	7.2
	Associate Degree	17	5.1
	Bachelor’s Degree	266	79.4
	Graduate	25	7.4
	PhD	3	0.9
**Previously Working in a Pandemic Hospital**	Yes	105	31.3
	No	230	68.7
**Department**	Intensive Care Unit	105	31.3
	Emergency Service	39	11.6
	Other’s	191	57.1
**Receiving on Mpox Disease Training**	Yes	14	4.2
	No	321	95.8
**Source of Mpox Disease Training** (***N* = 14)**	University	4	28.6
	Onthejob training	10	71.4
**Chronic Disease**	Yes	55	16.4
	No	280	83.6

The total mean score of the participants’ the Compliance with Isolation Precautions Scale was 79.37 ± 10.01. The mean scores of the subscales of the Compliance with Isolation Precautions Scale were as follows: Route of Infection subscale, 22.41 ± 3.21; Practitioner-Patient Safety subscale, 26.11 ± 4.37; Environmental Safety subscale, 17.49 ± 2.39; and Hand-Hygiene/Glove Use subscale, 13.18 ± 2.04 (Table 2).

**Table 2:** Scores of the compliance with isolation precautions scale, epidemic anxiety scale and perceived stress scale (*n* = 335)

Scales and subscales	Mean ± SD	Min-Max*
**Compliance with Isolation Precautions Scale**		
Route of Infection Subscale	22.41 **±** 3.21	5–25
Practitioner-Patient Safety Subscale	26.11 **±** 4.37	6–30
Environmental Safety Subscale	17.49 **±** 2.39	4–20
Hand-Hygiene/Glove Use Subscale	13.18 **±** 2.04	3–15
Total	79.37 **±** 10.01	18–90
**Epidemic Anxiety Scale**		
Epidemic Subscale	16.08 **±** 6.24	7–35
Economic Subscale	4.81 **±** 2.21	2–10
Quarantine Subscale	12.19 **±** 4.21	4–20
Social Life Subscale	16.02 **±** 5.61	5–25
Total	49.16 **±** 15.44	18–90
**Perceived Stress Scale**	26.39 **±** 7.76	0–56

* Minimum and Maximum score of the scales

The total mean score of the Epidemic Anxiety Scale among participants was 49.16 ± 15.44. The mean scores of the subscales of the Epidemic Anxiety Scale were as follows: Epidemic subscale, 16.08 ± 6.24; Economic subscale, 4.81 ± 2.21; Quarantine subscale, 12.19 ± 4.21; and Social Life subscale, 16.02 ± 5.61. The total mean score of the Perceived Stress Scale among participants was 26.39 ± 7.76 (Table 2).

There was a statistically significant difference in the total scores of the Compliance with Isolation Precautions Scale among nurses based on gender (t = 3.137; *p* =.002), previous experience working in a pandemic hospital (t = 2.148; *p* =.033), and their department (F = 4.086; *p* =.018). Additionally, there was a statistically significant difference in the total scores of the Epidemic Anxiety Scale among nurses based on gender (t = 1.989; *p* =.049). A statistically significant difference was also found in the total scores of the Perceived Stress Scale among nurses based on their chronic disease (t = 3.144; *p* =.002) (Table 3).

**Table 3:** Nurses’ scores of the compliance with isolation precautions scale, epidemic anxiety scale and perceived stress scale according to personal and working status (*n* = 335)

Parameters		Scale compliance with isolation precautions	Epidemic anxiety scale	Perceived stress scale
		Mean ± SD	Mean ± SD	Mean ± SD
**Gender**	Female	80.21 ± 10.21	50.12 ± 14.99	26.77 ± 7.58
	Male	76.33 ± 8.65	45.67 ± 16.67	24.98 ± 8.29
t		3.137	1.989	1.610
p		0.002*	0.049*	0.111
**Educational Level**	Vocational High School	79.58 ± 11.24	50.27 ± 16.41	26.85 ± 10.53
	Associate Degree	82.07 ± 7.06	52.68 ± 19.79	25.00 ± 9.48
	Bachelor’s Degree	79.08 ± 10.18	49.08 ± 15.29	26.65 ± 7.48
	Graduate	81.00 ± 8.23	46.00 ± 13.56	24.80 ± 7.01
	PhD	76.00 ± 13.11	59.50 ± 6.36	22.66 ± 5.03
KW		3.111	2.396	2.468
p		0.539	0.663	0.650
**Previously Working in a Pandemic Hospital**	Yes	81.13 ± 9.54	47.53 ± 14.69	26.93 ± 8.38
	No	78.57 ± 10.14	49.88 ± 15.74	26.16 ± 7.49
t		2.148	-1.285	0.772
p		0.033*	0.200	0.441
**Department**	Intensive Care Unit	81.34 ± 7.31	49.34 ± 14.51	27.01 ± 7.78
	Emergency Service	76.15 ± 14.53	50.45 ± 17.08	27.47 ± 8.05
	Other’s	78.96 ± 9.96	48.82 ± 15.69	25.84 ± 7.68
F		4.086	0.173	1.139
p		0.018*	0.841	0.321
**Receiving on Mpox Disease Training**	Yes	81.08 ± 8.54	47.27 ± 24.33	24.42 ± 10.58
	No	79.31 ± 10.07	49.23 ± 15.09	26.48 ± 7.62
Z		− 0.721	− 0.382	-1.065
p		0.471	0.702	0.287
**Chronic Disease**	Yes	81.32 ± 7.92	51.81 ± 16.66	29.26 ± 7.07
	No	78.97 ± 10.35	48.65 ± 15.18	25.83 ± 7.78
t		1.857	1.266	3.144
p		0.067	0.210	0.002*

t = t-Test, Z = Mann-Whitney U, KW = Kruskal-Wallis, F = One-way ANOVA, *p < 0.05

There was a statistically significant weak negative correlation between the score of the Compliance with Isolation Precautions Scale and Epidemic Subscale (r =-.124; p =.031) and Economic Subscale (r = −.129; p =.023). There was a statistically significant moderate positive correlation between the total scores of the Perceived Stress Scale and the total scores of the Epidemic Anxiety Scale (r =.399; p <.001). Also, a statistically significant moderate positive correlation was found between their Epidemic Subscale (r =.323; p <.001) and Economic Subscale (r =.316; p <.001) and Quarantine“(r =.337; p <.001) and Social Life (r =.332; p <.001) (Table 4).

**Table 4:** Correlation analysis (*n* = 335)

Scales and subscales		1	2	3	4	5	6	7	8	9	10
1. Compliance with Isolation Precautions Scale	*r*	1									
	*p*										
2. Route of Infection Subscale	*r*	0.846^**^	1								
	*p*	**< 0.001**									
3. Practitioner-Patient Safety Subscale	*r*	0.867^**^	0.567^**^	1							
	*p*	**< 0.001**	**< 0.001**								
4. Environmental Safety Subscale	*r*	0.784^**^	0.625^**^	0.532^**^	1						
	*p*	**< 0.001**	**< 0.001**	**< 0.001**							
5. Hand-Hygiene/Glove Use Subscale	*r*	0.785^**^	0.599^**^	0.594^**^	0.516^**^	1					
	*p*	**< 0.001**	**< 0.001**	**< 0.001**	**< 0.001**						
6. Epidemic Anxiety Scale	*r*	− 0.067	− 0.039	− 0.111	− 0.016	0.005	1				
	*p*	0.248	0.489	0.051	0.784	0.929					
7. Epidemic Subscale	*r*	− 0.124^*^	− 0.096	− 0.145^**^	− 0.048	− 0.092	0.840^**^	1			
	*p*	**0.031**	0.087	**0.009**	0.385	0.097	**< 0.001**				
8. Economic Subscale	*r*	− 0.129^*^	− 0.099	− 0.178^**^	− 0.045	− 0.024	0.789^**^	0.626^**^	1		
	*p*	**0.023**	0.075	**0.001**	0.413	0.662	**< 0.001**	**< 0.001**			
9. Quarantine Subscale	*r*	− 0.023	0.023	− 0.087	0.027	0.044	0.832^**^	0.520^**^	0.610^**^	1	
	*p*	0.681	0.677	0.120	0.620	0.431	**< 0.001**	**< 0.001**	**< 0.001**		
10. Social Life Subscale	*r*	0.021	0.021	− 0.011	0.030	0.085	0.870^**^	0.552^**^	0.610^**^	0.705^**^	1
	*p*	0.720	0.712	0.849	0.594	0.128	**< 0.001**	**< 0.001**	**< 0.001**	**< 0.001**	
11. Perceived Stress Scale	*r*	− 0.095	− 0.066	− 0.094	− 0.044	− 0.067	0.399^**^	0.323^**^	0.316^**^	0.337^**^	0.332^**^
	*p*	0.098	0.248	0.097	0.434	0.235	**< 0.001**	**< 0.001**	**< 0.001**	**< 0.001**	**< 0.001**

Note: Pearson’s correlation test. p values in bold indicates statistical significance. Abrreviation: r, correlation coefficient. *p <.05; **p <.01

## Discussion

The study found that nurses’ epidemic anxiety levels were moderate. Additionally, it was determined that a large majority of nurses (95.8%) had not received any formal training on Mpox. Nurses’ perceived stress levels increased in parallel with their epidemic anxiety. A statistically significant, though weak, negative correlation was observed between the Compliance with Isolation Precautions Scale and both the Epidemic and Economic subscales of the Epidemic Anxiety Scale. Conversely, a statistically significant moderate positive correlation was found between the Perceived Stress Scale and the Epidemic Anxiety Scale.

Following the emotional burden represented by the ongoing SARS-CoV-2 pandemic, the Mpox outbreak has emerged as a new source of threat and stress. It is well-known that healthcare workers experienced significant stress during previous COVID-19 pandemic [20, 21]. During the most recent SARS-CoV-2 pandemic, healthcare workers were reported to be emotionally affected. Various studies have shown that healthcare workers were at risk of psychiatric disorders during the COVID-19 pandemic due to multiple reasons [22, 23]. The geographic spread and re-emergence of Mpox disease have led to increasing concerns within society [24, 25]. In the current study, it was determined that nurses had moderate levels of epidemic anxiety. Additionally, nurses with chronic illnesses experienced higher levels of perceived stress compared to those without. During epidemic processes, nurses are in direct contact with patients carrying infectious diseases, which increases their risk of contracting infections. Concerns about their personal health, the presence of chronic illnesses, and the health of their loved ones may heighten their anxiety levels. Research on Mpox is quite limited, and healthcare workers have restricted knowledge about this disease. As nurses strive to cope with new and unfamiliar diseases, they often face uncertainties regarding applicable treatment protocols, the modes of disease transmission, and protective measures. This uncertainty can further elevate their anxiety levels [26]. Healthcare workers involved in patient care are at risk of infection, as transmission can occur through contact. Nurses may have experienced anxiety due to the risk of transmission, despite taking necessary precautions to protect themselves against the virus. Additionally, a combination of epidemic anxiety, workload, insufficient resources, societal pressures, and personal health concerns has heightened perceived stress. Therefore, providing psychological support, enhancing training programs, and ensuring safe working conditions for healthcare workers will help them cope with anxiety and stress more effectively.

Previous studies have shown that healthcare workers often lack adequate knowledge about Mpox. For example, a study evaluating the knowledge and attitudes of Bangladeshi nurses toward Mpox reported that 57.97% of the 1,047 participants demonstrated good knowledge [10]. In a survey study conducted with 606 healthcare workers, approximately two-thirds of whom were doctors (*n* = 204, 33.7%) or nurses (*n* = 190, 31.4%), only 4 out of 11 questions about Mpox were answered correctly by more than 70% of participants. Physicians were found to have a higher level of knowledge about Mpox compared to other groups. In the same study, 50.2% of participants reported confidence in diagnosing based on current Mpox diagnostic tests, while confidence levels for managing (38.9%) and diagnosing (38.0%) Mpox cases based on their existing knowledge and skills were reported to be low [27]. In the current study, the vast majority of nurses (95.8%) reported that they had not received any formal training on Mpox. While this suggests limited formal education on the topic, it does not necessarily reflect their overall knowledge, as nurses may have accessed information through other sources such as media, colleagues, or clinical guidelines. Therefore, it is not possible to evaluate nurses’ knowledge about Mpox based on the data obtained in this study. Perceived stress has increased in parallel with anxiety levels associated with the epidemic. These results are consistent with the study conducted by Sadek et al. [25] which found that more than half of the nurses experienced high levels of stress, and more than a quarter reported moderate levels of stress. This situation is likely linked to heavy workloads, previous experiences, fear of infection, and societal stigma surrounding the COVID-19 pandemic.

Mpox disease can be transmitted through human-to-human contact, inhalation of respiratory droplets, or contact with the skin lesions of an infected person. The disease can be controlled through measures such as vaccination, hygiene practices, and isolation or quarantine. It is anticipated that Mpox disease will have effects similar to COVID-19, but with challenges such as significant stigma, as social distancing and the visible skin lesions caused by the mpox virus could create additional difficulties [28, 29]. Healthcare workers faced similar challenges during the COVID-19 pandemic. In the current study, it was found that a weak negative correlation was found between nurses’ epidemic anxiety and economic concerns and their compliance with isolation precautions. In a study by Acar et al., which examined nurses’ fear of COVID-19 and their level of compliance with isolation, it was reported that as nurses’ fear of COVID-19 increased, their compliance with isolation also increased [30]. In our current study, this finding appears to be different. It can be said that this result is due to the lack of knowledge about the Mpox outbreak among nurses and their uncertainty about what actions they should take in this regard.

In this study, female nurses demonstrated higher compliance with isolation measures and reported greater anxiety levels. These gender-specific patterns may be attributed to psychosocial factors that have been consistently discussed in the literature. Female healthcare workers have been shown to exhibit higher levels of empathy, which may enhance their sensitivity to patient well-being and adherence to protective behaviors. Similarly, in previous studies, it was determined that female comply with isolation measures more than male and experience more anxiety [31–33]. Additionally, research indicates that women tend to perceive infectious disease threats as more serious compared to men, potentially increasing both their compliance and anxiety levels [34]. These interrelated factors highlight the need to consider gender as a significant determinant when evaluating healthcare workers’ behavioral and emotional responses during health crises.

An important finding of our study was that nurses working in the intensive care unit had higher compliance with isolation precautions compared to those working in emergency departments and other units. Additionally, those who had previously worked in a COVID-19 pandemic hospital showed higher compliance with isolation precautions compared to those who had not. Another study also reported that nurses working in intensive care units had higher compliance with isolation precautions compared to those working in other units [32]. The intensive care team’s frequent exposure to patients with infections, their continuous updating of infection control knowledge, and their experience with protective measures for patients in the intensive care unit during the COVID-19 pandemic can be considered possible reasons. It can be inferred that nurses’ experience and participation in ongoing in-service training are determining factors in their compliance with isolation precautions.

### Limitations and strengths of the study

This study has several limitations. Nurses were recruited from a single hospital in one region of the country, which may limit the generalizability of the findings to the wider nursing population. Data were collected using a self-administered questionnaire, which raises the possibility of social desirability bias influencing participants’ responses. However, the questionnaire was anonymous, and no identifying information was collected, which likely helped reduce this risk. While nurses working in the emergency department—a potentially high-risk area for Mpox—were included, other high-risk departments such as dermatology and infectious diseases were not analyzed separately due to limited sample sizes. Future research with larger and more diverse samples is recommended to better assess differences between departments in Mpox-related risk perception and preparedness. Additionally, the cross-sectional design restricts the ability to establish causal relationships. Despite these limitations, the study provides valuable insights into nurses’ levels of anxiety about the Mpox outbreak, their perceived stress, and their compliance with isolation precautions.

## Conclusions

The study found that nurses’ epidemic anxiety levels were moderate. However, the vast majority (95.8%) had not received any formal training on Mpox, highlighting a significant gap in outbreak-specific preparedness. Notably, female nurses and those with chronic diseases reported significantly higher levels of stress and anxiety. Additionally, nurses with prior experience working in COVID-19 pandemic hospitals and those employed in intensive care units showed higher compliance with isolation precautions.

A weak negative correlation was observed between compliance with isolation precautions and both epidemic anxiety and economic concerns, suggesting that increased anxiety may slightly reduce adherence to protective measures. Furthermore, a moderate positive correlation between epidemic anxiety and perceived stress underscores the psychological burden nurses experience during outbreaks. Based on the findings of this study, there is a clear need to strengthen nurses' preparedness for outbreaks like Mpox, particularly among those working in non-critical care settings and lacking prior outbreak experience. Targeted training programs on emerging infectious diseases should be implemented to reduce uncertainty and enhance compliance with infection control protocols. Mental health support must also be prioritized, especially for nurses with chronic illnesses or high stress levels. Tailored services such as counseling and peer support can help alleviate psychological strain. Embedding regular in-service training and simulation exercises—especially in departments like emergency units—can improve both theoretical and practical preparedness. Furthermore, proactive psychological support systems should be established, with specific attention to female staff who reported higher levels of anxiety. Improving working conditions through policy measures—such as ensuring adequate staffing, PPE availability, and fair compensation—remains essential to support nurses and sustain healthcare resilience during outbreaks.

## Data Availability

The data sets generated during and/or analysed during the current study are available from the corresponding author on reasonable request.
